# Structure and Dynamics of Oxidized Lipoproteins In Vivo: Roles of High-Density Lipoprotein

**DOI:** 10.3390/biomedicines9060655

**Published:** 2021-06-08

**Authors:** Hiroyuki Itabe, Naoko Sawada, Tomohiko Makiyama, Takashi Obama

**Affiliations:** Division of Biological Chemistry, Department of Pharmaceutical Sciences, School of Pharmacy, Showa University, 1-5-8 Hatanodai, Tokyo 142-8555, Japan; ns20170630@gmail.com (N.S.); t-maki@pharm.showa-u.ac.jp (T.M.); obama@pharm.showa-u.ac.jp (T.O.)

**Keywords:** atherosclerosis, oxidized LDL, oxidized HDL, acute myocardial infarction, lysoPC

## Abstract

Oxidative modification of lipoproteins is implicated in the occurrence and development of atherosclerotic lesions. Earlier studies have elucidated on the mechanisms of foam cell formation and lipid accumulation in these lesions, which is mediated by scavenger receptor-mediated endocytosis of oxidized low-density lipoprotein (oxLDL). Mounting clinical evidence has supported the involvement of oxLDL in cardiovascular diseases. High-density lipoprotein (HDL) is known as anti-atherogenic; however, recent studies have shown circulating oxidized HDL (oxHDL) is related to cardiovascular diseases. A modified structure of oxLDL, which was increased in the plasma of patients with acute myocardial infarction, was characterized. It had two unique features: (1) a fraction of oxLDL accompanied oxHDL, and (2) apoA1 was heavily modified, while modification of apoB, and the accumulation of oxidized phosphatidylcholine (oxPC) and lysophosphatidylcholine (lysoPC) was less pronounced. When LDL and HDL were present at the same time, oxidized lipoproteins actively interacted with each other, and oxPC and lysoPC were transferred to another lipoprotein particle and enzymatically metabolized rapidly. This brief review provides a novel view on the dynamics of oxLDL and oxHDL in circulation.

## 1. Introduction

### 1.1. Atherosclerotic Lesion

Atherosclerosis is a pathological condition in coronary arteries, aorta, and other vasculature, which leads to vascular events such as acute myocardial infarction (AMI). Atherosclerosis is characterized by enlargement of the intima and narrowing of the arterial lumen.

**Figure 1:**
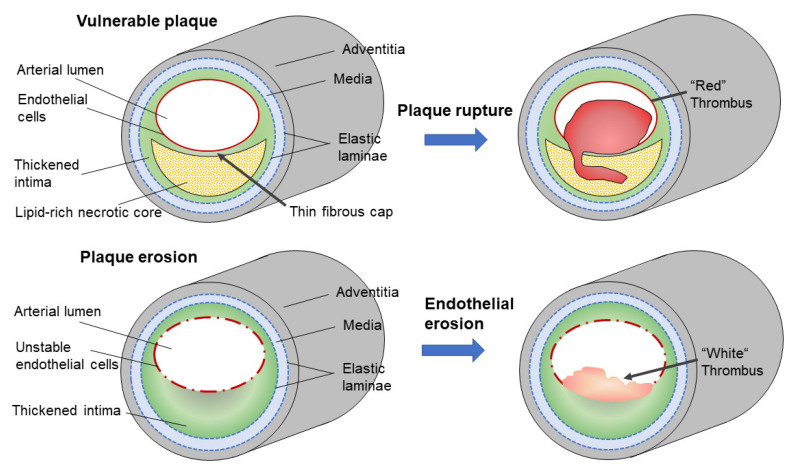
Two types of culprit lesions responsible for vascular events. Vulnerable plaque is characterized by a necrotic core, which accumulates a large amount of lipids covered with a thin “fibrous cap”. Inflammatory responses render the fibrous cap fragile and lead to plaque rupture. Once the fibrous cap is broken, a rapid formation of thrombus and fibrin occurs, and a red thrombus is formed. Plaque erosion, also called superficial erosion, is characterized by a thickened intima enriched in glycosaminoglycans, but with less lipid accumulation. Inflammatory responses make the endothelial cells unstable and lead to detachment of the cells. After the endothelial cells are lost, thrombus formation occurs, and a white thrombus is formed.

There are two types of atheromatous lesions that relate to these vascular events (Figure 1) [1]. Vulnerable plaques are atherosclerotic lesions containing large amounts of lipids and a number of lipid-accumulating foam cells that are covered with thin fibrous caps. Accumulation of macrophages and other types of leukocytes induce inflammatory responses and, eventually, the secretion of metalloproteinases, which could make the fibrous cap physically fragile; thus, such plaques have a high risk of being ruptured. Plaque ruptures induce acute thromboembolic responses containing blood components, which subsequently form red thrombi.

The other type of lesion is plaque erosion, also called superficial erosion, and it is characterized by thickened intima enriched with glycosaminoglycans, a small amount of lipid accumulation, and few macrophages [2]. Endothelial cells in this type of lesion may be delineated from the surface by yet unproven mechanisms. One possibility that has emerged recently is the involvement of neutrophil extracellular traps (NETs). A thrombus formed at the site of the eroded plaque contains leukocytes enriched with myeloperoxidase (MPO); neutrophils release proteolytic enzymes, MPO, together with DNA strings upon the formation of NETs [3]. However, there is little information on the contribution of lipoproteins to plaque erosion; and human arterial tissue is slightly different from that of athero-protective small animals [4], hence, this review focuses on the vulnerable plaques in human atherosclerosis.

### 1.2. Background on the oxLDL Hypothesis

Multiple factors contribute to the development of atherosclerotic lesions, such as low-density lipoprotein (LDL)-cholesterol levels, diabetes mellitus, hypertension, oxidative stress, and infectious diseases. In addition to these conditions, oxidative modification of lipoproteins has been recognized as a risk factor for atherosclerosis [5,6]. The possible importance of oxidized LDL (oxLDL) on atherogenesis has received attention since the 1980s, when a series of scavenger receptors for phagocytic uptake of oxLDL by macrophages were identified, such as scavenger receptor type A (SR-A), CD36, or lectin-like oxidized LDL receptor-1 (LOX-1) (refer to reviews [7,8]). Subsequently, localization of oxLDL in atherosclerotic lesions, especially in foam cells, were verified by immunohistochemical analysis [9,10,11]. The oxLDL hypothesis was an attractive possibility that explains the onset of atherosclerotic lesion formation for three reasons. First, it explains the correlation between plasma cholesterol levels and risk of cardiovascular disease, as suggested by epidemiological studies. Second, LDL receptor-dependent uptake of cholesterol cannot explain the enhanced development of atherosclerosis in patients with familial hypercholesterolemia (FH) who suffer from juvenile atherosclerosis despite a lack of functional LDL receptors. However, massive accumulation of cholesterol in macrophage-derived foam cells in patients with FH could be rationalized by scavenger receptors [12]. Finally, oxidative modification of LDL produces a large variety of oxidized lipids and modified peptides that are likely to promote endothelial injury and inflammatory responses [13,14,15].

A variety of oxidized products, including oxidized polyunsaturated fatty acids (PUFA), short-chain aldehydes, and oxidized phosphatidylcholine (oxPC), are formed in oxLDL prepared in vitro, many of which are found in plasma or in atherosclerotic lesions [16,17]. Molecular structures of oxPC formed in oxLDL were analyzed and some of them were identified [18,19,20,21]. In addition to oxidized phospholipids, some of the oxidatively modified apolipoprotein B-100 (apoB) peptides in atherosclerotic lesions were analyzed and identified using the liquid chromatography-tandem mass spectrometry (LC-MS/MS) technique [22,23,24,25].

Many studies were conducted on the pathological significance of in vivo oxLDL present in human circulation. In the mid-1990s, several groups developed sandwich ELISA procedures for the measurement of plasma oxLDL levels; studies using these immunological methods have shown the presence of oxLDL in human circulation (refer to reviews [26,27,28,29]). Large-scale clinical studies have proved the potential benefit of the measurement of circulating oxLDL levels as a marker for the risk of atherosclerosis [30,31]. Since the plasma concentration of LDL affects oxLDL levels directly, the ratio of oxLDL and apoB or oxLDL and LDL-cholesterol showed better clinical significance than simply estimating oxLDL concentration in plasma [28].

An LDL particle consists of one molecule of a large protein, apoB, and a variety of lipid molecules including phosphatidylcholine (PC), free cholesterol, cholesterol ester, and triacylglycerol (TG). Oxidative modification of such a multi-molecular particle produces complex and heterogenous modified forms of LDL. Consequently, it is a challenging issue to elucidate on the structural and metabolic characteristics of in vivo oxLDL present in circulation.

## 2. Oxidative Modification of In Vitro oxLDL

**Figure 2:**
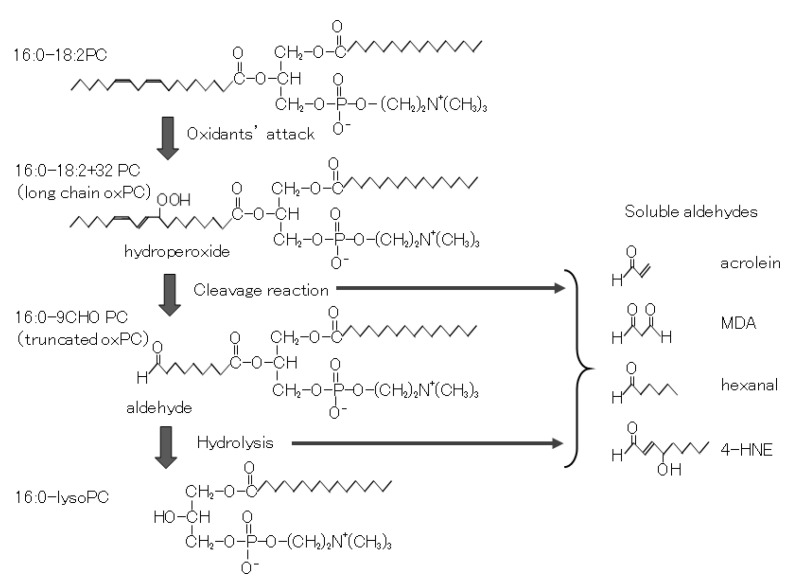
Cascade of oxidation products formed during oxidation of phosphatidylcholine (PC). PUFA moiety of PC is a target of the oxidation reaction. With the addition of oxygen to PUFA, PC hydroperoxides are formed. Often PC hydroperoxides are cleaved to form truncated oxPC and fragmented soluble aldehydes. The truncated oxPCs are subsequently hydrolyzed either by enzymes or by nonenzymatic reactions.

Among the constituents of LDL, hydrophobic lipids, cholesteryl ester, and TG are packed inside of the particle, and the hydrophobic core is surrounded by phospholipids, free cholesterol, and apoB. When LDL is oxidized in vitro, lipidomic analysis of oxLDL showed that PUFA-containing PC species are major targets of oxidation reaction [32,33,34]. PC species containing PUFA chains generate their hydroperoxide derivatives (PCOOH), which are subsequently converted into truncated PC products and small aldehyde fragments. Finally, the truncated PC is hydrolyzed to form lysoPC by either enzymatic or nonenzymatic pathways [32,33,34,35] (Figure 2). Cholesterol and TG are also oxidized to form various derivatives, such as oxidized fatty acids and 7-keto-cholesterol [36,37]. Reagents used for oxidation of LDL include copper sulfate, ferrous sulfate, and radical initiators such as 2,2′-azobis (2-amidinopropane) dihydro-chloride, lipoxygenase, or MPO. Oxidized products formed in LDL and modified by these oxidants partly overlap, but some products are selectively formed depending on the oxidants. For example, treatment of LDL with lipoxygenase produces mainly PCOOH, while incubation of LDL with a low concentration (e.g., mmol/L order) of copper sulfate produces several truncated oxPC and lysoPC [32]. During oxidative modification of lipoproteins, reactive aldehydes such as acrolein, malondialdehyde, and 4-hydroxynonenal bind to apolipoproteins to form various adducts at certain amino acid residues, mostly at lysine and histidine [23,24]. In addition, various amino acid residues, such as methionine and tryptophan, are oxygenated. MPO-dependent oxidation yields chlorinated or nitrated tyrosine residues in apoB protein together with oxidized lipids [38].

The formation of OxPC-protein adduct was demonstrated earlier [39,40]; however, structural analysis of OxPC-modified peptides was technically difficult to perform in earlier studies. Chemical modification procedures using methylamine and the advancement of LC-MS/MS instruments have helped to solve this problem [41,42]. As a result, precise structures of apolipoproteins, modified with oxPCs, have been reported recently [43,44].

A minimally modified LDL (MM-LDL), a type of oxidatively modified LDL with unique characteristics compared to copper-induced oxLDL, was prepared by soaking LDL in a dialysis bag in a buffer containing a low concentration of iron sulfate at 4 °C for 2–3 days [45]. Modification of the apoB protein was minimal, but contained significant amounts of oxPC production [46,47]. MM-LDL is reported to stimulate endothelial cells effectively [48], however, it is not a good ligand of macrophage scavenger receptors.

## 3. Clinical Evidence of oxLDL in CVD

As mentioned above, the measurement of plasma oxLDL has been performed for more than a quarter of a century, and mounting evidence has shown the clinical significance of increased plasma oxLDL levels in patients with acute coronary syndrome, cardiovascular diseases (CVD), diabetes mellites, and patients receiving hemodialysis (refer to reviews [26,27,28,29]). Many studies have reported oxLDL levels and oxLDL/LDL-cholesterol ratios in patient groups were significantly higher than those in control groups.

It has been noted that the plasma oxLDL level increased transiently in the acute phase after myocardial infarction or immediately after vascular injury by percutaneous transluminal coronary angioplasty (PTCA) treatment [49,50,51,52]. The source of oxLDL in the rapid increase in plasma is the oxLDL accumulated in the ruptured atherosclerotic lesions. Since clearance of oxLDL from circulation is very rapid [53], and the increase in the plasma oxLDL level is transient, oxLDL can be released rapidly from the lesions when vulnerable plaques rupture. In addition, in a controlled diet study using cynomolgus monkeys, even in the absence of plaque rupture, the plasma levels of oxLDL increased or decreased in correspondence to the progression or regression of the atherosclerotic lesions, respectively [54]. A transient increase in plasma oxLDL levels was also observed in apoE-knockout mice a few weeks before atherosclerotic lesion enlargement [55]. Treatment of rabbits with pre-established atherosclerotic lesions with probucol and atorvastatin decreased not only aortic lesion sizes, but also the contents of oxLDL deposited in the lesions [56]. These observations suggest that oxLDL can be transferred between circulation and the atherosclerotic lesions.

## 4. The Presence of oxHDL and Its Possible Function

It is well recognized that high-density lipoprotein (HDL) is an anti-atherogenic lipoprotein. There are several unique functions of HDL that make it athero-protective [57,58,59]. First, HDL acts as a carrier of reverse cholesterol transport that facilitates regression of atherosclerotic lesions. Second, HDL carries a few enzymes that hydrolyze oxidized lipids to inhibit propagation of lipid peroxidation; namely, paraoxonase-1 (PON1), lipoprotein associated-phospholipase A_2_ (Lp-PLA_2_), and lecithin-cholesterol acyl transferase (LCAT). Thirdly, the major protein of HDL, apoA1, acts as an antioxidant to protect LDL from oxidative modification. Since apoA1 is susceptible to oxidation, it reacts with oxidized centers formed in apoB or scavenges reactive oxygen species (ROS) before attacking apoB. It was reported that Met112 is highly reactive to oxidants and modification of this methionine residue reduced cholesterol efflux capacity [60]. After biotin-labeled oxPC was incubated with human plasma, the proteins conjugated with oxPC were collected and analyzed by LC-MS/MS. The most frequently detected target protein of oxPC was found to be apoA1 [42]. Finally, recent studies suggested that HDL acts as a carrier of sphingosine 1-phosphate (S1P), where S1P binds to one of the HDL proteins, apolipoprotein M (apoM). Subsequently, the apoM-bound S1P attenuates inflammation and apoptosis in vasculature and maintains the endothelial barrier function [61,62].

Modified structures of oxHDL have been studied, as in the case of oxLDL. Lipid peroxidation products, such as small aldehydes, can form adducts with apolipoprotein A1; in addition, oxygenated amino acid residues, nitrated or chlorinated tyrosine, and carbamylated lysine residues are also formed [60,63,64]. Furthermore, oxPC adducts of apoA1 were detected and analyzed [42,43,44]. Oxidation of HDL alters the functions of HDL. Modified HDL, either by copper-induced oxidation or treatment with acrolein, reduced cholesterol transport activity of HDL [65,66]. Oxidation of Met112 or chlorination of Tyr192 of apoA1 by MPO-dependent modifications reduced its cholesterol efflux capacity [60,63]. In addition, oxHDL increased LOX-1 expression in endothelial cells [67] and decreased migration of macrophages [68], which are thought to be regulated through NF-kB dependent pathways.

The presence of natural antibodies against oxidized apoA1 was reported earlier, suggesting the presence of oxHDL under physiological conditions [69]. To date, several groups have developed sandwich enzyme-linked immunosorbent assay (ELISA) procedures for the measurement of oxHDL in human circulation [70,71,72]. Using these assay systems, the increase in plasma oxHDL levels was shown to correlate with CVD [73], coronary artery calcification in patients with hemodialysis [74], metabolic mal-condition in obesity [75], and hyperlipidemic patients [76]. These observations suggest, in addition to oxLDL, that oxHDL is present in circulation and that oxHDL may correlate with those conditions. In some studies, mAbs recognizing, site-specific modifications of apoA1 were introduced. An ELISA system using a mAb recognizing sulfoxide derivative of Met112 was reported [77]. Another mAb that recognized 2-hydroxy-Trp72 in apoA1 was utilized to detect modified apoA1 in human plasma and in arterial plaques [25].

These observations suggest that oxHDL could be another biomarker for CVD, however, they seem to be confusing since HDL plays a role as anti-atherogenic lipoprotein and oxHDL may be produced due to the scavenging of harmful oxLDL. To answer this question, further understanding of the physiological function, clearance from circulation, and behavior in the plaques of oxidatively modified lipoproteins is critically needed.

## 5. Candidates for In Vivo oxLDL

During research on atherosclerosis, various types of in vitro oxLDL have been extensively utilized [78,79,80]. At the same time, efforts to separate and characterize the oxidized lipoproteins present in vivo have been continued [81,82]. Since the amount of oxLDL present in circulation is very small and may have a heterogeneous nature, it is difficult to isolate oxLDL from circulation. However, characterization of a variety of subfractions of LDL was carried out and provided useful information for understanding the heterogeneity of lipoproteins.

Hirano et al. found a subfraction of LDL enriched in patients with CVD or diabetes, which is called small dense LDL (sdLDL) [83,84]. When human plasma is mixed with heparin and either magnesium or manganese ions, most of the apoB-containing lipoproteins, i.e., normal LDL, VLDL, and chylomicron, form aggregates. After the aggregates are removed by centrifugation, HDL and a subclass of apoB-containing lipoproteins, with a higher density (1.044 < *d* < 1.063) and a smaller diameter (<25.5 nm) than average LDL particles, are recovered in the supernatant. The plasma concentration of sdLDL showed a good correlation with atherosclerotic burden [85]. Small dense LDL is thought to be more susceptible to oxidative modification partly because it interacts with proteoglycans in the vessel wall tissues [86]. Oxidative modification of the sdLDL fraction in patients administered atorvastatin was significantly reduced, judging by TBARS and LOOH assays [87]. However, extensive structural analysis of sdLDL particles has yet to be reported.

There is a possibility that lipoprotein small a (Lp(a)) is the expected oxLDL. Lp(a) is a unique subfraction of LDL-related lipoproteins. A soluble protein, apo(a), secreted from the liver, binds to the apoB protein with a disulfide bond to form an Lp(a) particle. The soluble protein apo(a) consists of multiple repeats of the kringle domain that is also present in the plasminogen activator inhibitor-1 (PAI-1) [88]. Tsimikas et al. clearly demonstrated that Lp(a) acted as a carrier of oxPC, since the kringle KIV_10_ domain in the apo(a) protein selectively interacted with oxPC molecules [89]. Patients with CVD showed increased plasma Lp(a) levels concomitant with the oxPC and apoB ratio when compared to control subjects [90]. In a number of epidemiologic studies, Lp(a) levels were suggested as a potential predictive marker for the risk of future CVD [91]. An unsolved issue is the genetic variance of apo(a) and its correlation with the risk of coronary heart disease (CHD). The kringle domain KIV_2_ is repeated many times in an apo(a) protein and the repeat numbers range from 1 to more than 40, since the number of exons corresponding to the KIV_2_ domain in the *APO(A)* gene varies individually [88]. The number of kringle domains inversely associated with plasma Lp(a) concentrations and the frequencies of short apo(a) proteins differ among ethnic groups. A meta-analysis of 40 studies demonstrated that individuals with smaller apo(a) isoforms have an approximately two-fold higher risk for CHD or ischemic stroke than those with larger apo(a) [92]. However, it was noted that the correlation between apo(a) and CHD risks is often documented in European populations, but not in Asian populations, suggesting there might be other factors to be considered [93].

LDL was divided into several subfractions using anion-exchange chromatography, due to its electronegative property, and the most electronegative fraction, LDL(−), was shown to be enriched with oxysterols [94]. Subsequently, the electronegative fraction (also called LDL5) was reported to have stimulatory properties against various cells, including endothelial cells and platelets [94,95,96,97,98]. An LDL(−) fraction recovered from normolipidemic subjects induced monocyte chemoattractant protein-1(MCP-1) secretion from human umbilical endothelial cells [95]. By contrast, an LDL(−) fraction recovered from ST-elevation myocardial infarction (STEMI) patients activated platelets and induced aggregation [96]. Moreover, LDL(−) stimulated human monocyte-derived macrophages to induce IL-1β release via inflammasome activation [98]. In addition, LDL(−) was reported to be enriched with free fatty acids, which may explain its electronegative feature [99]. Epidemiological observations showed that the LDL(−) subfraction was increased in patients with CVD, diabetes, renal disease, or non-alcoholic steatohepatitis (NASH), and, in many cases, medication of such patients decreased LDL(−) levels [100].

## 6. Involvement of oxHDL in In Vivo oxLDL Formation

**Figure 3:**
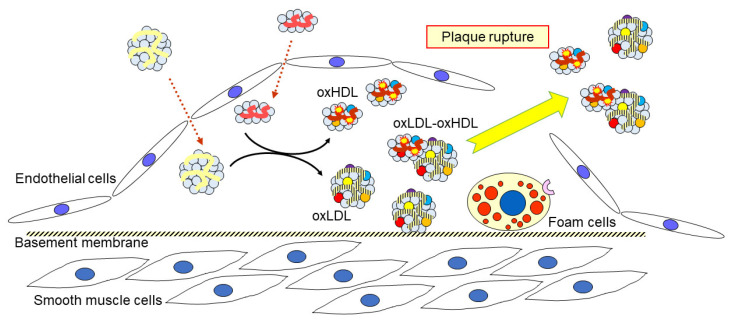
A proposed behavior of oxidized lipoproteins in atherosclerotic lesions and circulation. When LDL is oxidatively modified, HDL can interact with oxLDL to attenuate oxidative modification of LDL; in turn, HDL is modified to form oxHDL. A part of oxHDL may be associated with oxLDL. These oxidatively modified lipoproteins accumulate in vulnerable plaque and are released into the circulation when the plaque ruptures.

An anti-oxPC monoclonal antibody (mAb) was used to capture oxLDL in human plasma in our sandwich ELISA system [10]. We could not successfully isolate oxLDL from human plasma using immunoprecipitation strategies; however, we found that the LDL(−) fraction, separated on anion-exchange chromatography, was enriched in oxLDL. The apoB in the LDL(−) fraction was covalently modified with oxPC, as demonstrated by western blotting with the anti-oxPC mAb [101]. Surprisingly, the LDL(−) fraction contained a large number of HDL particles, which were detected using an agarose-gel electrophoresis and verified by electron micrographs. LC-MS/MS analysis revealed that the apoA1 in the LDL(−) fraction was highly modified by reactive aldehydes such as acrolein. More importantly, the oxLDL in the LDL(−) fraction was three times higher in the plasma of patients with AMI when compared to that of healthy controls. From these results, it can be predicted that a portion of oxLDL interacts with oxHDL in atherosclerotic lesions, which is then released into circulation when the lesions rupture (Figure 3). It is not yet clear how the oxHDL is associated with oxLDL; however, it is likely an electrostatic interaction, since they were recovered in the same fraction by ultracentrifugation, but were separated by agarose gel electrophoresis.

When the oxLDL-oxHDL interaction is considered, roles of HDL in oxidative modification of lipoproteins and its implication in CVD and other diseases should be reconsidered. As mentioned earlier, HDL is thought to be anti-atherogenic; however, the clinical significance of oxHDL and the oxLDL-oxHDL complex in several diseases, including CVD, needs to be further studied. One can speculate that oxHDL is readily generated as a result of scavenging reactive centers on oxLDL. If this is the case, the total oxidized lipoproteins, namely oxLDL plus oxHDL, would represent the strength of oxidative stress, and the ratio of oxHDL and oxLDL would suggest the protective efficacy of HDL from LDL oxidation.

It has been reported that the cholesterol efflux function of HDL decreases when it is modified by reactive aldehydes [65,68]. In addition, HDL obtained from patients with acute coronary syndrome had lower PON1 activity, more oxidative modifications on apoA1, and more MCP-1 gene expression in endothelial cells than HDL from normal subjects [102]. Thus, HDL diminishes the proatherogenic effect of oxLDL by reducing oxidative damages of oxLDL, while the anti-atherogenic function of HDL is also reduced since HDL itself is oxidized. In addition, oxidation of HDL may display pro-atherogenic functions through different mechanisms. Ru et al. reported that long-term infusion of oxHDL, dissolved in chitosan hydrogel, administered to LDL receptor knockout mice accelerated the progression of atherosclerotic lesion formation. In the oxHDL-infusion mice, regulatory T cells were reduced and Th17 cells were activated, which may have caused lesion development [103].

The structures and functions of oxHDL in circulation and those in atherosclerotic plaques may be different. Hazen et al. demonstrated that lipid-poor cross-linked apoA1 was enriched in plaques [104]. A site-specific modification, 2-hydroxy-Trp72 of apoA1, was shown to be abundantly present in atherosclerotic lesions, but not in circulation, and the modified apoA1 failed to accept cholesterol in an ABCA-1 dependent efflux assay [25].

The half-life of oxLDL and oxHDL seem to be different in circulation. The kinetics of oxLDL and oxHDL in human circulation have not been reported, however, it was shown previously that 95% of heavily oxidized LDL was cleared from the circulation within 10 min after being intravenously injected into rats [53]. By contrast, it took 24 h to reduce 95% of oxHDL from the circulation of Watanabe heritable hyperlipidemic (WHHL) rabbits [105]. These studies were conducted using copper-induced heavily modified lipoproteins; hence, further studies on in vivo kinetics using physiological types of modified lipoprotein particles, such as oxLDL-oxHDL complex, are required. Overall, at this point, it is difficult to evaluate the pathophysiological significance of oxHDL. Further investigation concerning systematic simulations, including the metabolic fate of oxHDL and the oxLDL-oxHDL complex, are critically needed to elucidate the function of HDL.

## 7. oxPC Metabolism in the Presence of HDL

**Figure 4:**
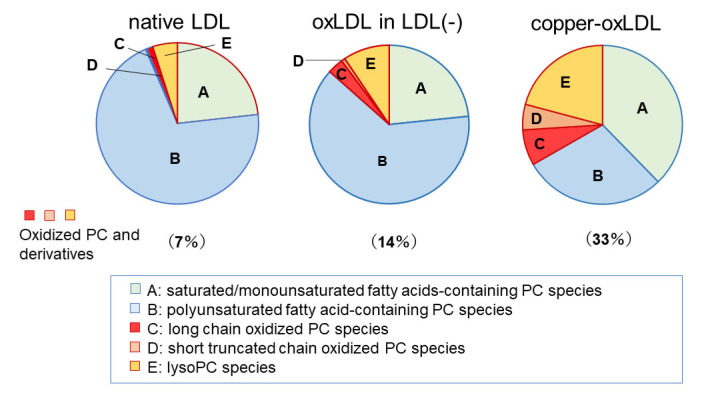
Composition of PCs in LDL, oxLDL recovered from LDL(−) fraction, and copper-induced oxidized LDL. The LDL(−) fraction was treated with anti-oxPC mAb to separate oxLDL from non-oxidized particles. PC molecular species were comprehensively analyzed by LC-MS/MS. More than 60 molecular species were classified into five groups: PC containing saturated and monounsaturated fatty acids (**A**), PUFA-containing PC (**B**), PC containing long chain oxidized fatty acids (**C**), oxPC containing truncated fatty acids (**D**), and lysoPC (**E**). The data are summarized from references [31,101].

It was noted that one of the characteristic features of the LDL(−) fraction is that accumulation of oxPC and lysoPC species is moderate, while its protein components, apoB and ApoA1, are extensively modified. When oxLDL is prepared in in vitro systems by incubation of LDL with copper sulfate for several hours, PUFA-containing PC species are consumed and lysoPC accumulates [32,33]. Compared with copper-induced oxLDL, which accumulates lysoPC to as much as 30% of total PC species, the change in PC composition in LDL(−) is very limited (Figure 4).

The contents of oxPCs and lysoPCs were not high in oxLDL in the LDL(−) fraction. This feature does not correspond with that of MM-LDL [45], a model of oxLDL enriched with oxPC, and with few apoB modifications. Thus, we should consider the environment of LDL during the oxidation reaction. In the treatment of LDL with some oxidation reagents in a test tube, which is a closed environment with a limited volume, hydrophilic reactive aldehydes, such as acrolein, would react with LDL in the same test tube. By contrast, since MM-LDL is prepared in a dialysis bag soaked in a large volume of buffer at 4 °C, lipids in LDL are oxidized gradually, and the hydrophilic reactive aldehydes produced are diffused through the dialysis membranes. In in vivo conditions, whether in tissues or in circulation, LDL is present along with other types of lipoproteins. An interaction between these particles may contribute to lipid transfer and lipid metabolism.

The behavior of oxPC and lysoPC in an oxLDL model was examined in the presence of HDL. LDL containing [^2^H]-labeled 1-palmitoyl-lysoPC was incubated with HDL for up to 4 h, then [^2^H]-labeled lipids in the LDL and HDL fractions were monitored using LC-MS/MS. Under these conditions, lysoPC in LDL was decreased during the incubation, while newly formed diacyl-PC in both LDL and HDL fractions increased concomitantly [106]. The production of diacyl-PC was abolished by an LCAT inhibitor. Exogenously added [^2^H]-labeled 1-palmitoyl-2-glutaroyl PC (PGPC), a short chain oxidized PC product, to LDL was destroyed within an hour unless an Lp-PLA_2_ inhibitor was added.

LysoPC molecules are not stable in membranes and are able to be spontaneously transferred to other membranes. Such a transfer of lysoPC between lipoproteins or liposomes was previously reported [107]. Intermembrane lipid transfer is not limited to lysoPC, and some oxidized lipids, including long-chain oxPC and oxidized cholesterol, can also move between lipoproteins [33]. From these observations, it is reasonable to consider that physicochemical transfer of lipids between lipoprotein particles and subsequent enzymatic actions of Lp-PLA_2_ and LCAT are important factors to fully understanding the formation of oxidized lipoproteins in vivo.

## 8. Conclusions

The roles of HDL in atherosclerosis and lipoprotein metabolism are more complicated than previously thought. Recent observations concerning the actions of oxHDL in vivo are accumulating; oxHDL is present in circulation and plaques, plasma oxHDL levels increase in patients with several diseases, and a part of oxLDL accompanies oxHDL in circulation. These findings could have a great impact on the classical view of HDL as anti-atherogenic agent. Although HDL is known to possess an overall anti-atherogenic property, HDL-LDL interactions and contributions of HDL oxidative modifications need to be further studied.

## Data Availability

Not applicable.

## Abbreviations

AMI	acute myocardial infarction
apoA1	apolipoprotein A1
apoB	apolipoprotein B-100
apoM	apolipoprotein M
apo(a)	apolipoprotein small a
CVD	cardiovascular diseases
ELISA	enzyme-linked immunosorbent assay
FH	familial hypercholesterolemia
HDL	high-density lipoprotein
LCAT	lecithin-cholesterol acyltransferase
LC-MS/MS	liquid chromatography-tandem mass spectrometry
LDL	low-density lipoprotein
LDL(−)	electronegative low-density lipoprotein
LOX-1	lectin-like oxidized low-density lipoprotein receptor-1
Lp(a)	lipoprotein small a
Lp-PLA_2_	lipoprotein associated phospholipase A_2_
mAb	monoclonal antibody
MCP-1	monocyte chemoattractant protein-1
MM-LDL	minimally modified low-density lipoprotein
MPO	myeloperoxidase
NETs	neutrophils extracellular traps
NF-kB	nuclear factor-kappa binding protein
oxHDL	oxidized high-density lipoprotein
oxLDL	oxidized low-density lipoprotein
oxPC	oxidized phosphatidylcholine
PC	phosphatidylcholine
PON1	paraoxonase-1
PTCA	percutaneous transluminal coronary angioplasty
PUFA	polyunsaturated fatty acid
sdLDL	small dense low-density lipoprotein
SR-A	scavenger receptor type A
STEMI	ST-elevation myocardial infarction
TBARS	thiobarbituric acid reactive substances
TG	triacylglycerol
WHHL	Watanabe heritable hyperlipidemic
